# Methicillin-Resistant Staphylococci and Macrococci at the Interface of Human and Animal Health

**DOI:** 10.3390/toxins13010061

**Published:** 2021-01-14

**Authors:** Karsten Becker

**Affiliations:** Friedrich Loeffler-Institute of Medical Microbiology, University Medicine Greifswald, 17475 Greifswald, Germany; karsten.becker@med.uni-greifswald.de

The global impact of methicillin-resistant *Staphylococcus aureus* (MRSA) clonal lineages on human and animal health continues, even considering the decreasing MRSA rates in some parts of the world [1,2]. Subsequent to the emergence of hospital-associated (HA) and community-associated (CA) MRSA, livestock-associated (LA) MRSA has become an additional threat for human and animal health, contributing significantly to morbidity, mortality and socio-economic costs [3,4,5]. Linking human, animal and environmental health, the basically old – but for a long time too little considered – holistic “One Health” concept is more imperative than ever to solve the challenges due to microorganisms which cross the boundaries of ecosystems [6].

Many aspects of the genetic basis, origin, distribution, transmission, virulence profile and introduction into the health care systems of MRSA are still poorly understood. The genetic basis of their almost complete resistance to β-lactam antibiotics, the staphylococcal cassette chromosome *mec* (SCC*mec*), has been characterized as large mobile genetic elements harboring not only the methicillin resistance-encoding genes but also genes mediating resistance towards other antibiotics, heavy metals and metalloids. This co-possession may lead to co-selection effects [7,8,9] that can negatively influence MRSA prevention efforts, for example, in husbandry [10].

The continued detection of novel SCC*mec* types and subtypes, as well as the recent expansion of the *mec* “alphabet” by the detection of the *mecC*, *mecB* and *mecD* genes, reflects the flexibility of staphylococci and their relatives, the macrococci, to resist the selection pressures occurring in their environment, [11,12,13]. The identification of plasmid-borne methicillin resistance in macrococci and staphylococci adds further to the enormous complexity of the genetic organization of methicillin resistance in the *Staphylococcaceae* family [14,15,16]. Thus, MRSA are not to be understood monolithically. This huge diversity and the ongoing dynamics of the distribution of their clonal lineages [17] is challenging culture-based and molecular routine diagnostics, as well as epidemiological studies. Moreover, the impact of coagulase-negative staphylococci and coagulase-positive non-*S. aureus* complex species, as well as the role of macrococci as source for methicillin resistance-encoding genetic elements in *S. aureus* are only scantily investigated. The impact of virulence factors, particularly toxins, on the adaptation of *Staphylococcaceae* members to novel hosts and/or ecosystems is also widely unknown.

Focusing on methicillin resistance and toxins in *Staphylococcaceae*, the aim of this Special Issue, “Methicillin-Resistant Staphylococci and Macrococci at the Interface of Human and Animal Health”, was to gather data on basic, epidemiological, ecological and medical aspects on the interface between animal keeping, wildlife and putative other niches on one hand, and human and animal health on the other.

Livestock-associated (LA) MRSA belonging to Clonal Complex (CC) 398 are a prime example of an emerging clonal lineage that leads to significant burden for the human health care system after overcoming the host species barrier and becoming a zoonotic pathogen [3,4,18,19]. Especially in areas with a high density of pig farming, LA-MRSA isolates account for a substantial proportion (up to 30% or more) of all MRSA recovered from patients in those regions, even from patients with sepsis or other invasive infections [20,21,22]. The introduction of LA-MRSA, particularly CC398 isolates, into hospitals occurs via patients with occupational livestock exposure [23,24,25]. Here, Cuny et al. report on their cross-sectional study, which has been performed to answer the question whether those with occupational exposure to raw meat and to raw meat products are at particular risk of nasal colonization by LA-MRSA [26]. While reports on contamination of raw meat by LA-MRSA have been published [27,28], the occupational handling of raw meat was not found to be associated with the acquisition of LA-MRSA, as was examined in the investigation of nasal swabs from more than 600 butchers and cooks [26]. The authors concluded that, probably, the general population may be at an even lower risk of acquiring LA-MRSA via raw meat contact, particularly if good kitchen hygiene practice is followed.

Ribeiro et al. [29] have analyzed another putative risk factor for the acquisition of methicillin-resistant staphylococci (MR-S), i.e., unpasteurized milk used in the production of artisanal cheeses. Their study, comprising five Brazilian dairy farms, indicated cross-contaminations with MR-S during cheese production. Thus, employees working in milking and artisanal unpasteurized cheese production could contribute to the dissemination of MR-S species through incorrect product handling procedures.

At the beginning of the emergence of the novel LA-MRSA clonal lineages, their virulence and capacity to cause severe infections were questioned [3,30]. In this issue, Treffon et al. [31] address the virulence potential of LA-MRSA CC398 recovered from respiratory specimens of cystic fibrosis (CF) patients in comparison with hospital-associated (HA) MRSA and methicillin-susceptible *S. aureus* (MSSA) strains. CF patients are characterized by reduced lung function and life expectancy due to chronic bacterial airway infections, notoriously by *S. aureus*. The authors observed that LA-MRSA strains from CF patients were strongly hemolytic and more cytotoxic than HA-MRSA strains. The LA-MRSA strains were shown to be also more invasive than the MSSA isolates tested [31]. Of interest, their investigations also confirmed for LA-MRSA that that the adaptation of *S. aureus* to CF airways is an individual and very complex process which might hamper the success of anti-staphylococcal treatment of respiratory infections of this patient population.

From a basic clinical point of view, one could say that “an *S. aureus* is an *S. aureus* is an *S. aureus*,” which means that almost every *S. aureus* strain is able to cause the wide range of pyogenic infections for which this pathogen is notorious. However, some strains are “special” in the sense that they possess additional virulence factors, which are not part of the *S. aureus* core genome. These include isolates that possess specific toxins, such as the classical and newly described pyrogenic toxin superantigens (PTSAgs), and also single members of the synergohymenotropic toxin family, such as the Pantone–Valentine leucocidin (PVL). PVL-possessing isolates are frequently associated with skin and soft-tissue infections and can also cause a special, severe entity of pneumonia described as necrotic hemorrhagic pneumonia [32]. Mairi et al. [33] collected more than 2000 samples from humans, livestock, wild and companion animals, food products and the aquatic environment to study the distribution of MSSA and MRSA isolates in different ecological niches in Algeria and to determine the occurrence the prevalence of PVL gene-positive isolates. Of note, 10.6% of the detected *S. aureus* isolates tested positive for PVL and some of these co-harbored other toxin genes, such as exfoliative toxins or PTSAgs. All PVL-positive MRSA isolates belonged to the ST80-IV CA-MRSA clonal lineage. In contrast, PVL-positive MSSA isolates emerged from different sources. Another main finding of this study was the high prevalence of toxinogenic MSSA strains, mainly due to strains carrying the toxic shock toxin-1 (TSST-1) gene. Again, high genotypic diversity was found for strains tested positive for the TSST-1-encoding gene.

While MRSA-ST80, mostly represented by *spa* type t044, has become known as the predominant human CA-MRSA lineage throughout Europe, Northern Africa and the Middle East, food- and animal-associated isolates are rare. In a second article in this issue, Mairi et al. [34] summarize reports about positive MRSA-ST80 isolates in farm and wild animals and other ecological niches such as food. In their systematic review comprising PubMed-catalogued papers (*n* > 100) from 2003 to 2019, they found that, at least for this known PVL-positive lineage, the overall proportion has decreased in many countries in recent years.

Scholtzek et al. [35] characterized equine *S. aureus* isolates that show elevated minimal inhibitory concentrations (MICs) for oxacillin but could not be categorized as MRSA. Those so-called borderline oxacillin-resistant *S. aureus* (BORSA) strains pose a challenge for routine laboratories in both human and veterinary medicine if no special procedures and molecular confirmation are practiced. The term BORSA comprises a heterogeneous group of mostly weak oxacillin- or methicillin-resistant staphylococcal isolates that are still poorly understood and inadequately defined [36]. They have in common that they miss any kind of *mec* genes; thus, the additional penicillin-binding protein (PBP) PBP2a is not formed. Instead, they are characterized by hyper-production of beta-lactamases or their resistance is associated with point mutations of the PBPs occurring usually in *S. aureus*. By core genome multilocus sequence typing, Scholtzek et al. observed the close relatedness of the isolates belonging to either ST1 or ST1660 [35]. The beta-lactamase activity of the 19 isolates included was found to be associated with an inducible *blaZ* gene.

It is a truism that proper selection of the breeding line of an experimental animal and also their colonization status (microbiota) may drastically influence the outcome of a given experiment. Raafat et al. [37] report on their investigations into the molecular epidemiology of MSSA and MRSA in laboratory rats including both *Rattus norvegicus* and *Rattus rattus*. These investigations are of importance because rats can also serve as a reservoir for MSSA as well as HA- and LA-MRSA and other MR-S [38,39,40]. In a comparison of the colonization of laboratory rats with the composition of the natural *S. aureus* population in wild and captive rats, significant differences between the different populations were detected. While the natural *S. aureus* population of wild rats comprised mainly CC49- and CC130-MSSA strains, laboratory rats showed a lower nasal *S. aureus* carriage rate but were colonized with many different, mostly typically human-associated, *S. aureus* lineages. Common LA-MRSA (*spa* type t011) and other animal-associated MRSA lineages (CC30 and CC130) were found in wild and captive rats. The CC130 isolates possessed the *mecC* gene instead of *mecA*.

Since certain *S. aureus* lineages reveal substantial zoonotic potential, effective “One Health” concepts against the spread of MRSA have to take transmissions among farm animals, and also companion and wild animals and humans, into account. Especially, measures need to be intensified (i) to lower the resistance selection pressure by reducing consumption of antibiotics and co-selecting agents (e.g., metals as food supplements), (ii) to reduce the transmission of pathogens between animals and humans in husbandry by improving basic hygiene and (iii) to improve the surveillance of multi-resistant organisms.
